# Reading and lexical–semantic retrieval tasks outperforms single task speech analysis in the screening of mild cognitive impairment and Alzheimer's disease

**DOI:** 10.1038/s41598-023-36804-y

**Published:** 2023-06-15

**Authors:** Israel Martínez-Nicolás, Francisco Martínez-Sánchez, Olga Ivanova, Juan J. G. Meilán

**Affiliations:** 1 Faculty of Psychology, University of Salamanca, 37008 Salamanca, Spain; 2 Faculty of Psychology, University of Murcia, 30100 Murcia, Spain; 3 Faculty of Philology, University of Salamanca, 37008 Salamanca, Spain; 4 Institute of Neuroscience of Castilla y León, 37007 Salamanca, Spain

**Keywords:** Biomarkers, Neurological disorders

## Abstract

Age-related cognitive impairment have increased dramatically in recent years, which has risen the interes in developing screening tools for mild cognitive impairment and Alzheimer's disease. Speech analysis allows to exploit the behavioral consequences of cognitive deficits on the patient's vocal performance so that it is possible to identify pathologies affecting speech production such as dementia. Previous studies have further shown that the speech task used determines how the speech parameters are altered. We aim to combine the impairments in several speech production tasks in order to improve the accuracy of screening through speech analysis. The sample consists of 72 participants divided into three equal groups of healthy older adults, people with mild cognitive impairment, or Alzheimer's disease, matched by age and education. A complete neuropsychological assessment and two voice recordings were performed. The tasks required the participants to read a text, and complete a sentence with semantic information. A stepwise linear discriminant analysis was performed to select speech parameters with discriminative power. The discriminative functions obtained an accuracy of 83.3% in simultaneous classifications of several levels of cognitive impairment. It would therefore be a promising screening tool for dementia.

## Introduction

Automatic speech analysis is progressively becoming a promising tool for the screening of different mental disorders^1,2^ and nervous system pathologies^3,4^. This technique assumes that when speaking, we generate an acoustic signal which constitutes a direct behavioral measure of the processes necessary to produce language. The disruption of such processes would result in significant changes in various speech features which, when correctly identified, could be considered as biomarkers of specific pathologies^5,6^. The method of automatic speech analysis is becoming attractive because it allows to collect a sample, that is, the acoustic signal from the patient remotely, conveying them minimal costs and discomfort. These characteristics are in significant contrast to the neuropsychological batteries or neuroimaging tests, rather more costly in terms of time and money, and in many cases invasive for the patient.

The efficacy of speech markers analysis has been tested for the detection of such pathologies as depression^7,8^, schizophrenia^9^, concussion^10^ or Parkinson’s disease^11^. Yet, one of the fields in which the search for acoustic biomarkers is becoming particularly relevant is that relating to the discrimination and detection of Alzheimer’s disease (AD) and mild cognitive impairment (MCI)^12,13^.

AD is the most common cause of dementia^14^ accounting for between 60 and 80% of cases, a figure consistent across cultures^15^. In dementia due to Alzheimer’s disease, the insidious onset is generally related to memory complaints as the first symptom. In the course of AD, however, there is a steady decline of cognitive functioning that affects several cognitive domains, including language and speech. With regard to the latter, previous studies have found that AD affects acoustic, temporal, and prosodic features of speech. Speakers with AD present with flat prosody deriving from changes in shimmer, fundamental frequency, and formants^13,16,17^, with a higher number of pauses and voice breaks but with a lower speech rate^12,18^, and with very significant alterations in the speech rhythm^19^. Importantly, the literature using these parameters for AD detection is extensive and proves their great accuracy for group classification^20,21^.

MCI is defined as an objective cognitive deficit of insufficient severity to meet the criteria for dementia, and which does not cause functional problems in activities of daily living^22,23^. Although heterogeneous, this deficit is usually characterized by language impairments in various tasks, such as verbal fluency, naming, lexical decision^24^ or changes in spoken language^25^. Even in cases where there is no direct cognitive deficit of a linguistic type, as is the case of amnestic MCI, difficulties such as a weakening of syntax-semantics integration in complex sentence processing have been identified^26^. In speech production, people with MCI present with lower speech rate, longer hesitations^27^, and changes in fundamental frequency and formants^28^. With automatic analysis, these speech features have proved to be useful in the detection of MCI either by itself^18^, used in addition to neuropsychological tests^29^, and in combination with other linguistic measures^30, 31^.

Changes in speech parameters of people with AD or MCI would be directly related impairments in several cognitive processes whose performance is compromised in both conditions. Some disturbances in speech rhythm have been related to difficulties in lexical–semantic access, so that they are expected to appear when the speaker presents with difficulties in word-finding^32,33^. Thus, the increase in the number of pauses would be a compensatory mechanism necessary to correctly perform on an impaired process^34^. Syntax planning and semantic relationship between words are also suggested to affect speech rate^26,35, 36^. Indeed, the length and the degree of syntactic complexity of sentences are related to speech deficits reflecting an impairment in working memory and attention^37^. In conversation, speech deficits seem to appear due to the difficulties of people with AD or MCI to comprehend their interlocutor’s utterances, plan their answers, and anticipate turn-endings, reflecting an impairment of executive functions^38^. Impairments in memory also predict the occurrence of changes in speech, mainly increased frequency and variability of pauses in both story recall^34,39^ and autobiographical memory^40^. It is also proposed that cognitive load influences voice quality parameters^41,42^ and such measures of modulation of pitch and amplitude as jitter and shimmer^43^.

From the above it follows the type of the task used to elicit oral language is one of the factors directly affecting speech performance in AD and MCI. In fact, based on the comparison of speakers’ performance on different tasks, some studies suggest that tasks with greater cognitive load outperform others in predicting the clinical condition of the patient^44^.

Building on previous studies that used a reading task to develop automatic screening tools for AD and MCI^13,19^, our goal is to improve their prediction by using new tasks that compromise participants’ cognitive resources. In doing so, we use the already explored reading task coupled with a new task on semantic verbal fluency. In reading, people with AD show particularly more pauses and other speech disturbances when exposed to texts with low frequency words, possibly because of the impairment in their semantic memory. Their reading is also characterized as slow and with poor expressive prosody due to encoding difficulties^45^. With the verbal fluency task, we mean to engage older adults’ resources for several cognitive domains, including lexical access, semantic memory search, attention processes, and executive functions. In addition, compared to most works which perform binary group classification, we aim to obtain a combination of speech parameters that would allow screen between healthy older adults (HC), MCI, and AD. In addition, we will perform an analysis of each of the tests separately and together, so that we can test their efficacy and whether the combination provides an advantage in the assessment.

## Methods

### Participants

A total of 72 participants divided into three groups were recruited for the study. Of these, 24 were diagnosed with MCI following the criteria of the International Working Group on Mild Cognitive Impairment^46^. The remainder were 24 healthy older adults who formed the healthy control group, and 24 people with dementia of Alzheimer’s type, all of whom were selected from a larger pool of samples to match the participants with MCI in age and schooling.

All participants had to be native speakers of Spanish, with no history of drug or alcohol abuse, no history of psychiatric illness, no presence of severe sensory deficits that would preclude the administration of cognitive tests, and a minimal level of schooling years to have acquired literacy.

All participants signed the informed consent form. The study was conducted in accordance with the Declaration of Helsinki and its subsequent amendments, and the European Union regulations concerning medical research. This research received the approval of the Ethics Committee of the State Reference Centre for the Care of People with Alzheimer’s Disease and other Dementias (Salamanca, Spain), attached to the Spanish Ministry of Social Rights and 2030 Agenda.

HC participants and participants with MCI were recruited from attendees of the Psychological Attention Service for the Prevention of Cognitive Problems in the Elderly of the Municipal Psychosocial Support Unit of the Council of Salamanca and the University of Salamanca (Spain). Participants with AD were recruited from the State Reference Center for the Care of People with Alzheimer's Disease and other Dementias, where they were diagnosed by the Spanish National Health Service following the NIA-AA criteria^47^. Table 1 contains data from the groups.

**Table 1:** Sample descriptive data.

	HC	MCI	AD
Age (mean/SD)	82.5 (7.04)	84.125 (6.34)	81.25 (6.83)
Schooling years (mean/SD)	9.66 (3.29)	8.41 (3.76)	9.37 (3.65)
MMSE (mean/SD)	27.37 (2.2)	23.66 (3.05)	21.58 (3.22)

No differences were found between the three groups in age F_2,69_ = 1.096, *p* = 0.340 or schooling F_2,69_ = 0.764, *p* = 0.470. Groups expectedly showed significant differences in MMSE scores F_2,69_ = 25.144, *p* < 0.001, which were found between HC and MCI (diff = 3.7, *p* < 0.001), HC and AD (diff = 5.79, *p* < 0.001), and MCI and AD (diff = 2.08, *p* = 0.042).

### Instruments

Participants were diagnosed by a clinician after going through neuropsychological assessment by using Dem-Detect toolkit^48^.This toolkit includes normative studies on Spanish population for several test. It is structured in two sessions. First one includes Mini-Mental State Examination, Memory Impairment Screening, and verbal fluency. The second one consist of Free and Cued Reminding Test, Trail Making Test, and Boston Naming Test. Furthermore we used additional questionnaires for the assesment of depression and activities of daily living.

Speech and language recordings were made using an iPad and a head-mounted condenser microphone, MiC Plus from Apogee. These recordings were preprocessed to eliminate possible background noise and ensure quality, and then analyzed using Praat software, version 6.0^49^, in order to extract a wide set of parameters including acoustic, rhythm, and voice quality features.

### Speech analysis

The data set obtained from the recordings was preprocessed to increase the quality of the audio files before extracting speech features that could accurately describe the various aspects of the subjects' speech of interest in this study. The audio recordings contained both the participants' and the researcher's voices. Since only the participants' portions of the audio were of interest, the researcher's voice was manually removed after listening to the audio files. The recordings were also analyzed in search of static background noise and unexpected noise caused by sources such as opening doors. Subsequently, the background noise spectrum obtained from the silence regions of the audio files was removed from all files. Finally, all files were normalized, to avoid possible differences due to being recorded with different settings depending on the position of the speaker, the location of the microphone and the distance to the speaker. After the pre-processing stages, the audio files were saved as 16-bit mono-channel .wav files. The original sample rate of 44,100 Hz was retained uniformly throughout all the audio files.

Our analysis included those speech parameters that usually prove altered in reading tasks of people with MCI and AD. Thus, we have introduced duration parameters, such as phonation time, speech rate, and both number and duration of pauses. Since fluency and rhythm are pointed out as particularly disturbed in reading, we also introduced articulation rate and syllabic parameters (nPVI, rPVI, average duration). Spectral analysis parameters, such as fundamental frequency, formants and their bandwidths, asymmetry and center of gravity, are also usually altered in MCI and AD^50^. Previous studies also found intra and intersyllabic pitch trajectories (TrajInter, TrajIntra). We expected pauses and rhythmic parameters to be specially affected in semantic verbal fluency task. Furthermore, being the goal of this study to engage cognitive resources, which may be reflected in speech quality and spectral parameters, we also included jitter and shimmer, Acoustic Voice Quality Index (AVQI), Harmonics to Noise ratio (HNR), and band energy parameters (BE). Statistics from several parameters, i.e. mean, median, standard deviation, maximum, and minimum, were also used as speech parameters. A complete list of the features used in this analysis may be found in online Annex 1.

Since we expect the changes in speech parameters in each of the tasks to be due to the involvement of different processes, the audio files have been analyzed separately. Still, for exploratory purposes, the same speech parameters have been extracted from each of them.

### Procedure

In order to assign the participants to their respective group, each of them went through a cognitive assessment lasting three sessions of approximately one hour each, usually conducted one week apart. Session one and two consisted of anamnesis and DemDetect Toolkit, as described in the instruments section, while session three was used for additional tests if necessary and participation in other studies. This assessment was carried out regardless of wether the participant had a previous diagnosis made by the health system or not, in order to confirm and characterize their level of impairment.

In addition to the assessment, participants were asked to perform two tasks that were recorded. The first consisted of a reading task, based on the reading aloud the first two sentences of the novel *Don Quixote*. In our previous study^50^, we made a strong case for the choice of this text excerpt, allowing to control for semantic load due to the combination of high-frequent and low-frequent words, complex syntax, and relative encyclopedic control.

The second task consisted in orally repeating the sentence "*Parcheesi tiles are colored*…” and completing it with four words for expected colors: “*green, yellow, red, and blue*”. Parcheesi is a very popular game in the Spanish culture and is therefore part of the general encyclopedic knowledge. Thus, the task required participants to retrieve from their memory both the encyclopedic knowledge about the four colors of the Parcheesi tiles (semantic load), and the corresponding lemmas for these highly common color names (lexical load). We predicted that the advantage of this task over a classical semantic verbal fluency task relied on significantly reducing the variability of participants’ responses due to the limitation of the expected elicitations. We made sure that all participants were familiar with the game. Elicited utterances were analyzed regardless of whether the answers (i.e., the named colors) were correct or not.

### Statistical analysis

Statistical analysis was conducted using IBM SPSS Statistics for Windows, Version 26.0. The speech measures from both tasks were subjected to linear discriminant analysis with a stepwise method for the inclusion of variables. Diagnosis, i.e. HC, MCI or AD, were used as dependent variables. The analysis included a leave-one-out cross-validation.

In addition, we will perform an analysis of the two tasks separately using the same procedure in order to compare the performance of the classification using just one task.

## Results

### Combination of features from both tasks

Discriminant analysis yielded two functions. Function 1 (percentage of variance explained = 61.9%; eigenvalue = 2.396, canonical correlation = 0.840; Wilks’ lambda = 0.119, χ^2^ = 135.181, d.f. = 24, *p* < 0.001) contributes to discriminate between the three groups, finding a more pronounced difference between HC and MCI (see Table 2 presenting group centroids). Function 2 (percentage of variance explained = 38.1%; eigenvalue = 1.475, canonical correlation = 0.772; Wilks’ lambda = 0.404, χ^2^ = 57.543, d.f. = 11, *p* < 0.001) specifically contributes to differentiate AD group from the rest.

**Table 2:** Group centroids of the functions.

	Function 1	Function 2
HC	− 1.788	− 0.927
MCI	1.917	− 0.752
AD	− 0.129	1.678

The chi-square of both functions is statistically significant and therefore both contribute to a large extent to the correct classification of the three groups. The Wilks’ lambda close to 0 in Function 1 indicates that this function captures variability that can be attributed almost exclusively to differences between groups. Function 2 would also be capturing mainly inter-group variability, but it seems to be more influenced by intra-group differences. The large canonical correlations point at that the scores of both Function 1 and Function 2 have a strong relation with between-group differences.

According to the structure matrix and coefficients (Table 3), the predictive capacity of Function 1 is especially related to the fourth formant, voice quality parameters (BE 3750–4000, AVQI CPPS) and rhythm (rPVI). In the case of Function 2, a clearer influence of the quality parameters as well as syllabic variability are percieved. In both functions, most of the influence relies on the reading task. However, the influence of the reading parameters seems to be more marked in Function 1, while in Function 2 the influence is shared by both tasks. In accordance with these functions, 93.1% of participants were correctly classified. In the cross-validation study, this percentage was 83.3%. Table 4 provides precision, sensitivity and specificity for this classifier.

**Table 3:** Discriminating speech parameters.

Parameters	Wilks’ Lambda	Structure Matrix		Standardized discriminant function coefficient	
		Function 1	Function 2	Function 1	Function 2
P AVQI TILT dB	0.185	0.021	0.331	0.350	0.923
R BE 1000–1250 Hz	0.174	− 0.052	0.233	− 1.429	0.368
R BE 750–1000 Hz	0.209	0.137	0.236	1.801	0.308
R rPVI	0.158	0.182	0.270	0.608	0.551
R BE 3750–4000 Hz	0.144	0.213	− 0.173	0.469	− 0.301
R AVQI CPPS	0.217	0.194	− 0.236	1.532	− 0.796
P B3 SD	0.139	− 0.006	0.007	0.047	0.588
R AVQI JITTER ABS	0.168	− 0.101	0.079	1.231	− 0.150
R F4	0.164	0.215	0.054	0.723	0.050
P Average Syllable Duration	0.146	0.013	0.221	− 0.533	0.533
P BE 3250–3500 Hz	0.141	− 0.115	− 0.003	− 0.541	0.066
P TrajIntraZ	0.142	0.169	0.109	0.465	0.320

Parameters starting with R correspond to the reading task, and those starting with P, to the Parcheesi task.

**Table 4:** Precision, sensitivity and specificity for the classifier.

	Precision	Sensitivity	Specificity
HC	0.8	0.83	0.89
MCI	0.86	0.83	0.93
AD	0.86	0.83	0.91

### Analysis by task

First, the discriminant analysis using the features extracted from the parcheese task yielded two functions: Function 1 (percentage of variance explained = 74.1%; eigenvalue = 0.511, canonical correlation = 0.582; Wilks’ lambda = 0.562, χ^2^ = 38.942, d.f. = 8, *p* < 0.001) and Function 2 (percentage of variance explained = 25.9%; eigenvalue = 0.178, canonical correlation = 0.389; Wilks’ lambda = 0.849, χ^2^ = 11.078, d.f. = 3, *p* < 0.05). Group centroids for these functions can be found in Table 5. The functions use just four feautures: articulation rate, jitter absolute, AVQI tilt and Falls (see Table 6 for structure matrix and coefficients). These functions correctly classify the three groups with a 66.7% of accuracy, and 56.9% in the crossvalidation. Precision, sensitivity and specificity may be found in Table 7.

**Table 5:** Group centroids for the functions in the parcheese and reading task.

	Function 1	Function 2
Parcheese task		
HC	− 0.150	− 0.578
MCI	− 0.772	0.366
AD	0.922	0.212
Reading task		
HC	− 0.615	0.891
MCI	1.266	− 0.016
AD	− 0.650	− 0.875

**Table 6:** Wilk’s Lambda, structure matrix, and Standardized discriminant function coefficient of the features in the parcheese and reading task.

Parameters	Wilks’ Lambda	Structure matrix		Standardized discriminant function coefficient	
		Function 1	Function 2	Function 1	Function 2
Parcheese task					
Articulation rate	0.658	− 0.314	− 0.339	− 0.681	− 0.279
Jitter absolute	0.672	0.449	− 0.165	0.710	− 0.310
AVQI Tilt	0.677	0.491	0.468	0.616	0.587
Falls	0.682	− 0.297	0.727	− 0.552	0.796
Reading task					
Jitter local	0.419	− 0.143	0.127	0.793	0.683
Harmonics to Noise ratio (SD)	0.438	0.460	− 0.339	0.430	− 0.703
AVQI CPPS	0.516	0.436	0.157	1.302	1.076
BE 3750–4000 Hz	0.426	0.426	0.047	0.615	0.160
Coefficient of variation syllabic duration	0.413	0.165	− 0.485	0.677	− 0.293
rPVI	0.399	0.108	− 0.571	− 0.245	− 0.623

**Table 7:** Precision, sensitivity and specificity for the classifiers separate by task.

	Parcheese			Reading		
	Precision	Sensitivity	Specificity	Precision	Sensitivity	Specificity
HC	0.5	0.5	0.75	0.59	0.66	0.77
MCI	0.54	0.54	0.77	0.72	0.75	0.85
AD	0.7	0.7	0.85	0.7	0.58	0.87

For its part, in the reading task, we obtained two functions: Function 1 (percentage of variance explained = 60.7%; eigenvalue = 0.836, canonical correlation = 0.675; Wilks’ lambda = 0.353, χ^2^ = 69.216, d.f. = 12, *p* < 0.001) and Function 2 (percentage of variance explained = 39.3%; eigenvalue = 0.542, canonical correlation = 0.593; Wilks’ lambda = 0.648, χ^2^ = 28.811, d.f. = 5, *p* < 0.01) whose group centroids are found in Table 5. This classifier included jitter local, the standard deviation of harmonics to noise ratio, AVQI CPPS, BE 3750–4000 Hz, the coefficient of variation syllabic duration and rPVI (see Table 6 for structure matrix and coefficients). These functions together differentiate between the three groups with a 73.6% of accuracy, being this score 66.7% in the crossvalidation. Precision, sensitivity and specificity may be found in Table 7.

## Discussion

The main objective of this study was to obtain a combination of speech parameters that would allow to screen between healthy older adults and older adults with either MCI or AD. For that purpose, we examined a set of acoustic, rhythm, and voice quality parameters of speech while participants performed an oral reading task and a recall task involving semantic memory load. The results show that it is possible to successfully differentiate between the three groups relying on acoustic, speech quality, rhythm, and duration parameters. In addition, we tested the advantage of using more than one task involving different cognitive processes, so that the classifier combining parameters from two tasks is more effective.

The parameters obtained and described as significant in this study are well reported in the literature on the application of automatic speech analysis for non-invasive discrimination of pathological aging. Specifically, the significant variation in such parameters as distortions in formants^28^, alterations in AVQI, PVI and TrajIntra^51^, and syllable duration^52^ had been reported. The present study contributes with evidence on the relevance of changes in energy bands for group discrimination. To the best of our knowledge, this parameter has not been previously used in speech analysis of cognitive impairment, although other measures of spectral analysis were found to be altered^53^.

Much of the literature on speech analysis of cognitive aging focuses on the study of rhythm variables and, more fundamentally, on such temporal variables as pauses, understood as a reflection of the difficulties in performing some of the cognitive processes required by the language task. In contrast to our hypothesis that in the Parcheesi task the parameters of rhythm and duration, specifically pauses, would be more relevant for group discrimination, we found that these parameters are in the minority. Overall, we could observe only two significant rhythm parameters referring to duration and syllabic variability. These parameters were already identified in previous studies comparing speech changes in MCI and AD against HC^50^.

In the present work, however, we could identify a different series of speech parameters that point out such factors as cognitive load as differentiators. Furthermore, our result are in line with previous work arguing for the influence of executive functions on speech in healthy and pathological aging^54,55^. In this sense, our results support the assumption that the type of the elicitation task and its associated cognitive load is an important factor for improving the speech-based screening of dementias by jeopardizing the cognitive resources of older adults^44,56^.

One of most important contributions of this study is the performance of speech analysis comparing all three groups (HC, MCI, and AD) at the same time. The literature on the detection of these entities is extensive, but seems to coincide in achieving predictive algorithms with 90–95% accuracy for AD, and a lower accuracy of 75–85% for MCI^20,21^. Most of these studies are limited to discriminating between two groups^27,30^. Even when the samples include the three groups, they usually perform binary classifications^57^. In these cases, moreover, the performance of the classifications are similar to those found in our study with three categories. If we take as a reference some of the most outstanding studies such as toth 2018^27^, we will see that they achieve an accuracy, sensitivity and specificity lower than 0.75 in a binary classification of HC vs MCI; or the case of Fraser^30^ with figures that are closer to the result obtained here in which these statistics are around 0.83, only after combining speech parameters with other sources of information. On the other hand, AD detection in binary comparisons tends to show better results than those obtained here with accuracy, sensitivity and specificity usually above 0.90^58,59^. To our knowledge, there are only few other studies that have accomplished a similar multi-class analysis, such as the works by Gosztolya and colleagues^31^ and O'Malley and colleagues^60^ both which show an accuracy of 65% and sensitivity and specificity below 0.80. For their part, De Looze and colleagues^38^ and Bertini and colleagues^61^ found accuracy, sensitivity and specificity similar to ours in the range of 82–86%.

As a way of verification, we tested the performance of the speech tasks separately. In this sense, we were already building on our previous work, which focused on reading. Thus, we had obtained in binary classifications HC vs AD an accuracy of 92.4%^51^. Here, for a multiclass classification, we obtained a much lower accuracy of 66.7%, which is still higher than that of the new proposed task. It is with the combination of the two tasks that we observe that the classifier shows overall good results, improving the performance of the reading alone and allowing us to classify the three groups. We believe that this opens a door to continue exploring new tasks based on the available knowledge about the cognitive deficits shown by the target entities to be evaluated. This being applicable both to those proposed in this study and to the exploration of other pathologies with this same method of speech analysis. Some possibilities for MCI and AD are the aforementioned autobiographical recall, which would account for episodic memory deficits, or tests aimed more directly at engaging executive processes such as letter-number sequencing.

Arguably, the fundamental objective of speech-based analysis in cognitive aging is to differentiate MCI from HC and, furthermore, to identify those profiles which more likely will evolve to AD. More and more experimental studies are focusing on this objective in recent years^19,39^. However, there is a social need to develop and carry out screening of more advanced stages of cognitive impairment, since nearly 40% of aging population with such impairment remains undiagnosed^14,62^.

One of the most outstanding potentials of the results of speech analysis in cognitive aging lies in the possibility to perform such screening by means of electronic devices^51^, applications^44,63^ or even phone calls^64^. Although the sample for this research was collected in the laboratory context, it is easy to transfer the same speech collection protocol to other contexts without any loss of quality, even possibly doing so in noisy environments^65^. Importantly, the available evidence shows that cognitive screening based on speech collection and assessment is accepted and positively valued by users due to the simplicity of its administration^66,67^. Other practical advantages of this technique, like the objectivity of the evaluation process, the possibility to reach a wide population sector due to the widespread use of technologies (i.e., smartphones), and the consequent low burden for the healthcare system, can be highlighted^68^.

One of the limitations of this study relates to the cultural constraint of the elicitation task. As described above, the semantic verbal task is strongly based on the assessment of culturally conditioned knowledge of the Spanish society. Therefore, it might not be generalizable to many other contexts. Although Parcheesi is a universally known game, it can be much less popular in other cultures and it will be complex to adapt it as a normative task to different contexts. Moreover, it should be considered that although all participants of this study knew the game, it could be criticized that the mere knowledge of it does not imply either that they knew the exact colors or that, even knowing them, could produce a quick response unless the level of familiarity was high. Future research should explore whether the task is performed correctly, how many colors the participants are able to say, and if they are those corresponding to the game. This process could also be automated by means of automatic speech recognition systems^69^. On the other hand, we must bear in mind that given the number of parameters included in the joint analysis of the two tasks, it will be necessary to continue testing on larger samples.

## Conclusion

This study shows that automatic speech analysis can be used in the assessment of MCI and AD in cognitive aging. It particularly proves that research on speech-based detection of dementia (or any other disorder) should involve tasks that take into account the critical cognitive impairments of the target groups. This assumption led us to include and test a new elicitation task considering cognitive alterations observed in aging speakers when performing verbal fluency tests. As a result, we could obtain a good classification accuracy. This result invites us to continue its development in tests with larger samples and to consider the possibility of performing a clinical validation to consolidate it as a screening tool. We also expect to improve it by exploring new tasks compromising other cognitive domains in older people with MCI and AD. We believe that correctly classifying the three groups of older adults (HC, MCI, and AD) makes this combination of parameters a reasonable option for use in the clinical settings, given that it addresses several needs without diminishing the sensitivity to the MCI stage, i.e., the earliest and most likely to be undiagnosed in cognitive aging.

## Supplementary Information

Supplementary Information.

## Data Availability

The data that support the findings of this study are available from the corresponding author upon reasonable request.
