# Supplementary material for: Reading and lexical–semantic retrieval tasks outperforms single task speech analysis in the screening of mild cognitive impairment and Alzheimer's disease
Source: Sci Rep. 2023 Jun 15;13:9728. doi: 10.1038/s41598-023-36804-y (PMC10272227; doi:10.1038/s41598-023-36804-y)
Supplement: Supplementary file 1 — Supplementary Information. [file 41598_2023_36804_MOESM1_ESM.docx]

**ANNEX 1.** Complete list of features used in the analysis.

| Duration and rythm | Total duration, number of voice breaks, number of pauses, phonation time, speech rate, articulation rate. |
| --- | --- |
| Syllabic features | Average Sillable Duration and its standard deviation, coefficient of variation of sillable duration, rPVI, nPVI. |
| F0 | Minimum, maximum, range, mean, median, standard deviation, difference between maximum and minimum, difference between maximum and median, difference between minimum and median. |
| Amplitude | Minimum, maximum, range, mean, median, standard deviation, difference between maximum and minimum, difference between maximum and median, difference between minimum and median. |
| Center of gravity | Center of gravity and its standard deviation. |
| Jitter | Local, Relative Average Perturbation, local absolute, ppq5, ddp. |
| Shimmer | Locacl, local dB, apq3, apq5 apq11. |
| Harmonics to noise ratio (HNR) | HNR and its standard deviation, NHR. |
| Formants | F1 to F5 and their standard deviation, Bandwiths of the five formants and their standard deviation. |
| AVQI | AVQI, CPPS, HNR, NHR, shimmer loc, jitter loc, jitter abs, slope, tilt, center of gravity and standard dedviation, kurtosis. |
| Band Energy | 250, 500, 750, 1000, 1250, 1500, 1750, 2000, 2250, 2500, 2750, 3000, 3250, 3500, 3750. |
| intersyllabic pitch trajectories | Intrasyllabic, Intersyllabic and trajectory of phonation |
| Prosody | Gliss Rises Falls |
| Spectral features | Assymetry, kurtosis, tilt, autocorrelation |
